# Supplementary figures and images for: Maximally efficient prediction in the early fly visual system may support evasive flight maneuvers
Source: PLoS Comput Biol. 2021 May 20;17(5):e1008965. doi: 10.1371/journal.pcbi.1008965 (PMC8136689; doi:10.1371/journal.pcbi.1008965)

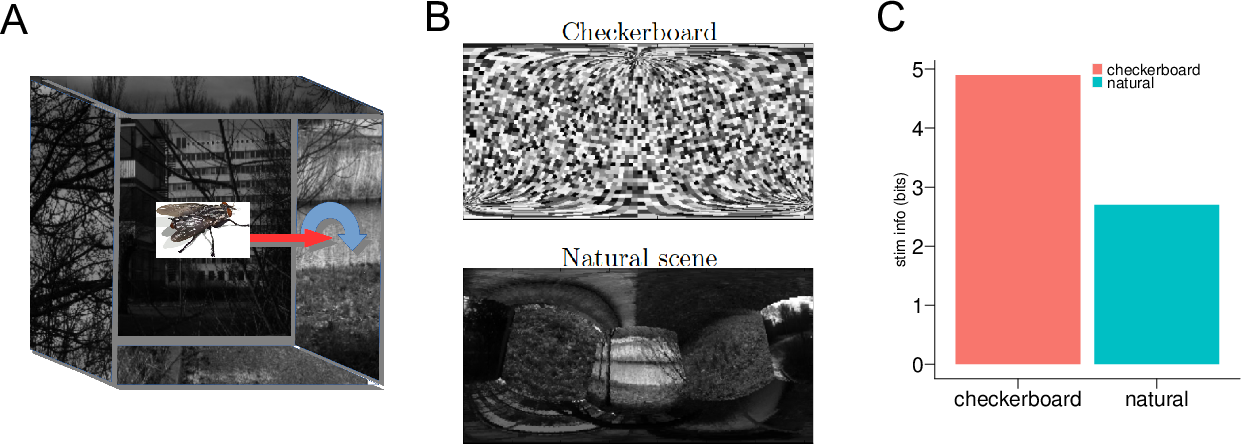

Supplement: S1 Fig — A) Schematic depiction of the visual stimuli for the simulation, recompiled from [46]. Six natural images (five are shown here, with one excluded to reveal the fly’s viewing perspective) were randomly selected from the van Hateren dataset [65]; each image was patched onto a different face of a cube. Assuming that the fly is located in the center of this cube, we obtain the visual experience of the fly’s ego-rotational motion by rotating this cage around a particular motion direction shown by the dark blue arrow. We then project the moving natural scene cage to a unit sphere that represents the fly’s retina, following the protocol introduced in [39, 45]. There are ∼5,500 local motion detectors (LMD) evenly distributed on this unit sphere. The responses of those LMDs whose locations are within a VS cell’s dendritic receptive field (Σazimuth = 15 and Σelevation = 60°, tiling along the fly’s anterior-posterior axis, see details in supplementary Materials and methods) are then integrated as the input current to this particular VS cell. B) Mercator maps with both checkerboard and natural scene backgrounds, at 1° resolution in spherical coordinates. C) ego-motion information inferable in checkerboard and natural scene backgrounds. The stimulus is a constant rotation of 500°/s from [46]. Note that there is only half of the information about this motion stimulus using the background of natural scene textures compared to the checkerboard background. (TIF) [file pcbi.1008965.s001.tif]

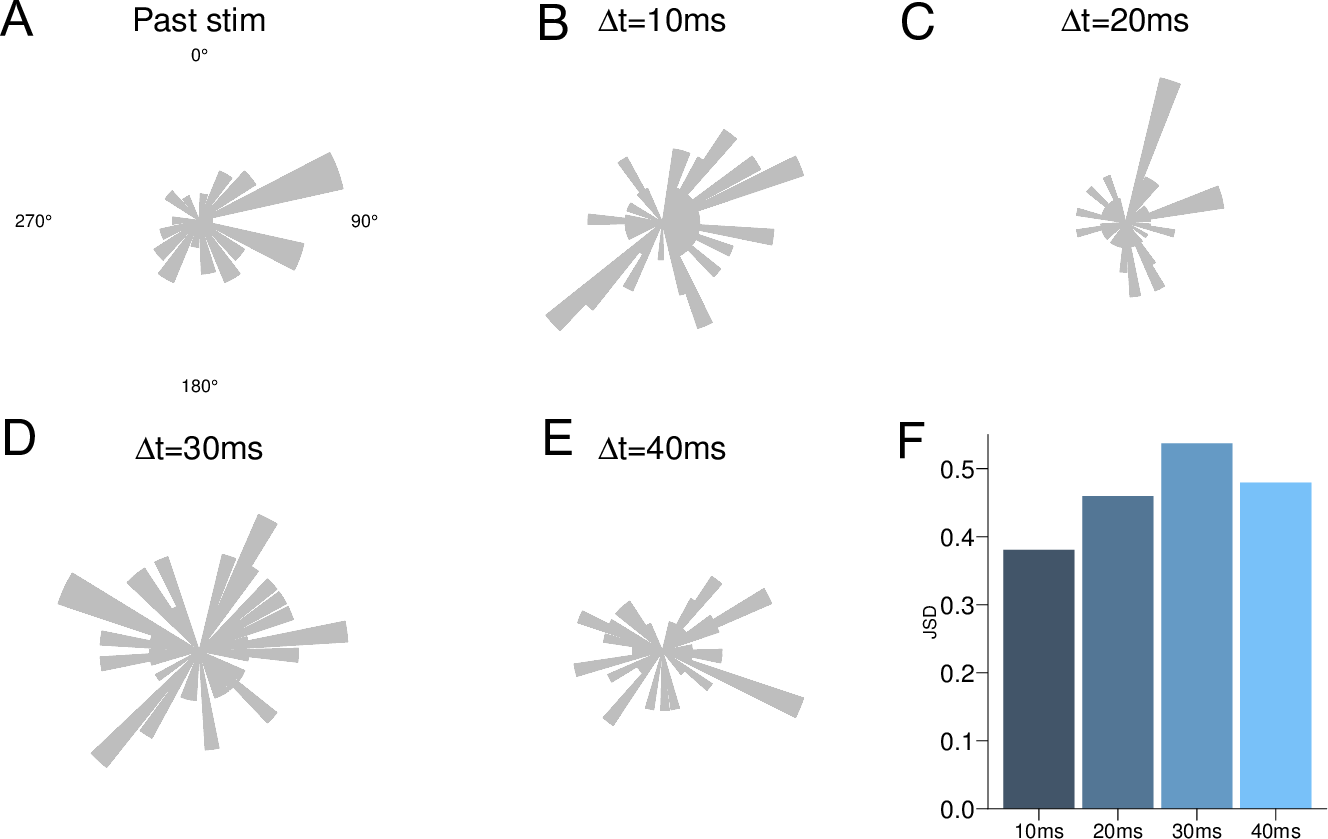

Supplement: S2 Fig — Egorotation distributions for different time steps during the evasive maneuver. Here we focus on the egorotations to which the VS network is sensitive. Because the VS network is only responsive to combinations of roll and pitch motions, i.e. motions within the fly’s coronal plane, we represent all stimuli with their corresponding vectors in this plane. A) The egorotation distribution at 10ms before the onset of evasive maneuvers. B) The future egorotation at 10ms after the initiation of evasive maneuvers. C) Similar to B, but for the egorotation at 20ms within the evasive maneuver. Here, most of the banked turns slow down and counter banked turns start.) D) Similar to B, but for the egorotation at 30ms within the evasive maneuver. This motion corresponds to the start of the counter-banked turn. E) Similar to B, but for egorotations a fly would experience at the end of the evasive maneuver. This motion corresponds to the slowing down of counter-banked turn and the completion of evasive maneuver. All of these egorotation distributions have comparable entropy ∼4 − 4.3 bits. F) The Jensen–Shannon divergence between the past egorotation distribution and the egorotations at Δt = 10, 20, 30, 40ms of evasive maneuvers, respectively. (TIF) [file pcbi.1008965.s002.tif]

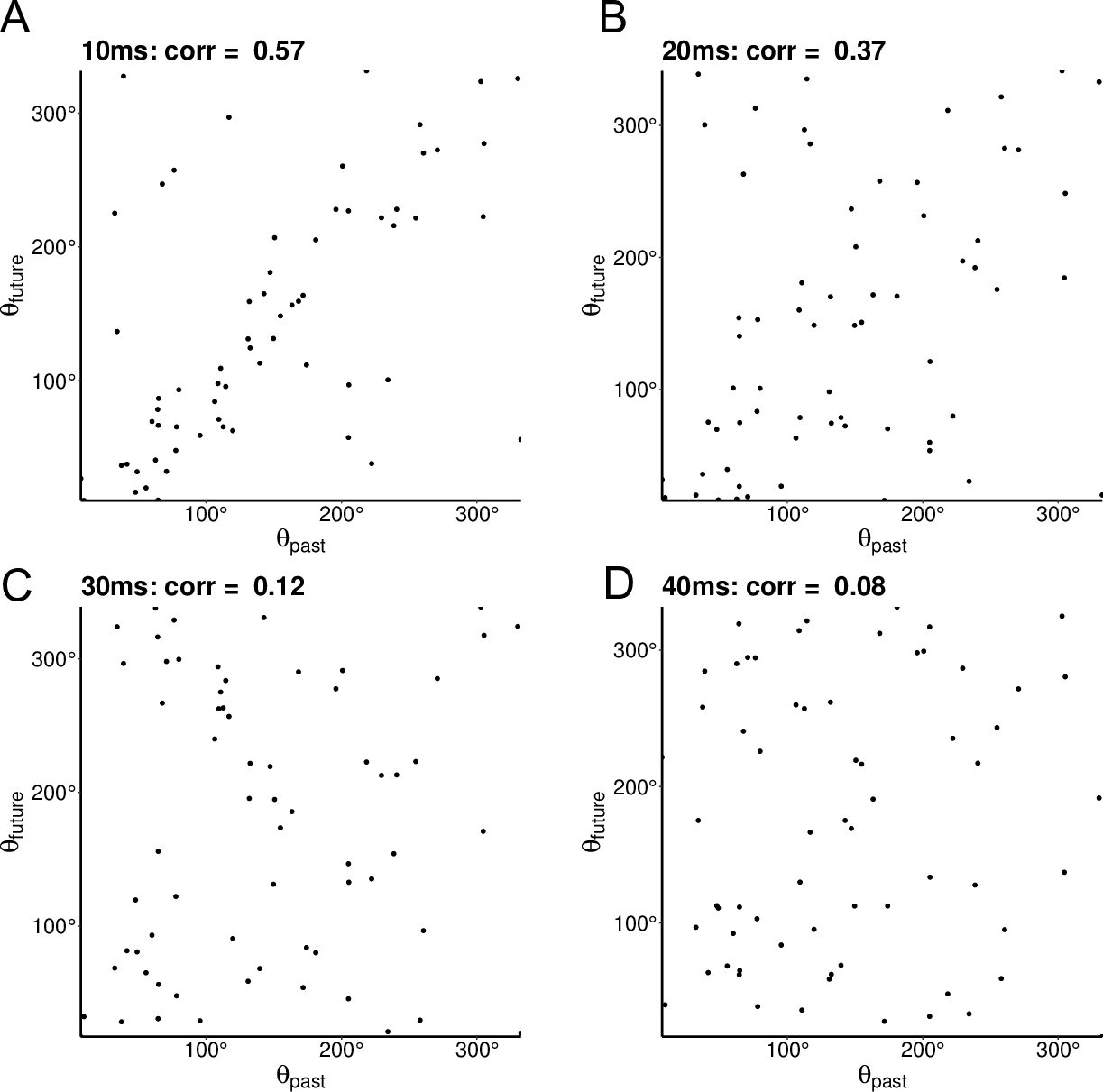

Supplement: S3 Fig — These egorotations are calculated as the axis of rotation, combining the rotational angles along both roll and pitch body axes. A) The correlation between the egorotation distribution at 10ms before the onset of evasive maneuvers and the egorotation distribution 10ms into evasive maneuvers. B) Similar to A), but for the egorotation distribution 20ms into evasive maneuvers. C) Similar to A), the egorotation distribution 30ms into evasive maneuvers. D) Similar to A), the egorotation distribution 40ms into evasive maneuvers. (TIF) [file pcbi.1008965.s003.tif]

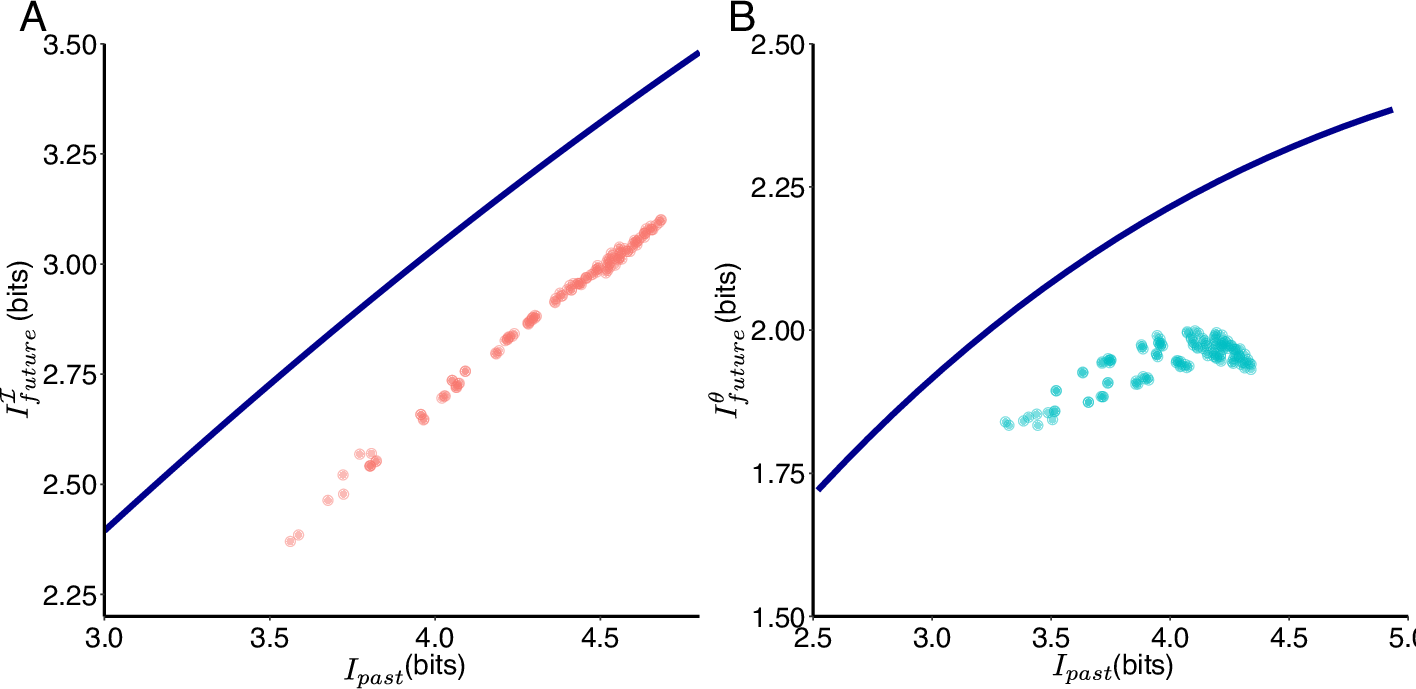

Supplement: S4 Fig — (TIF) [file pcbi.1008965.s004.tif]

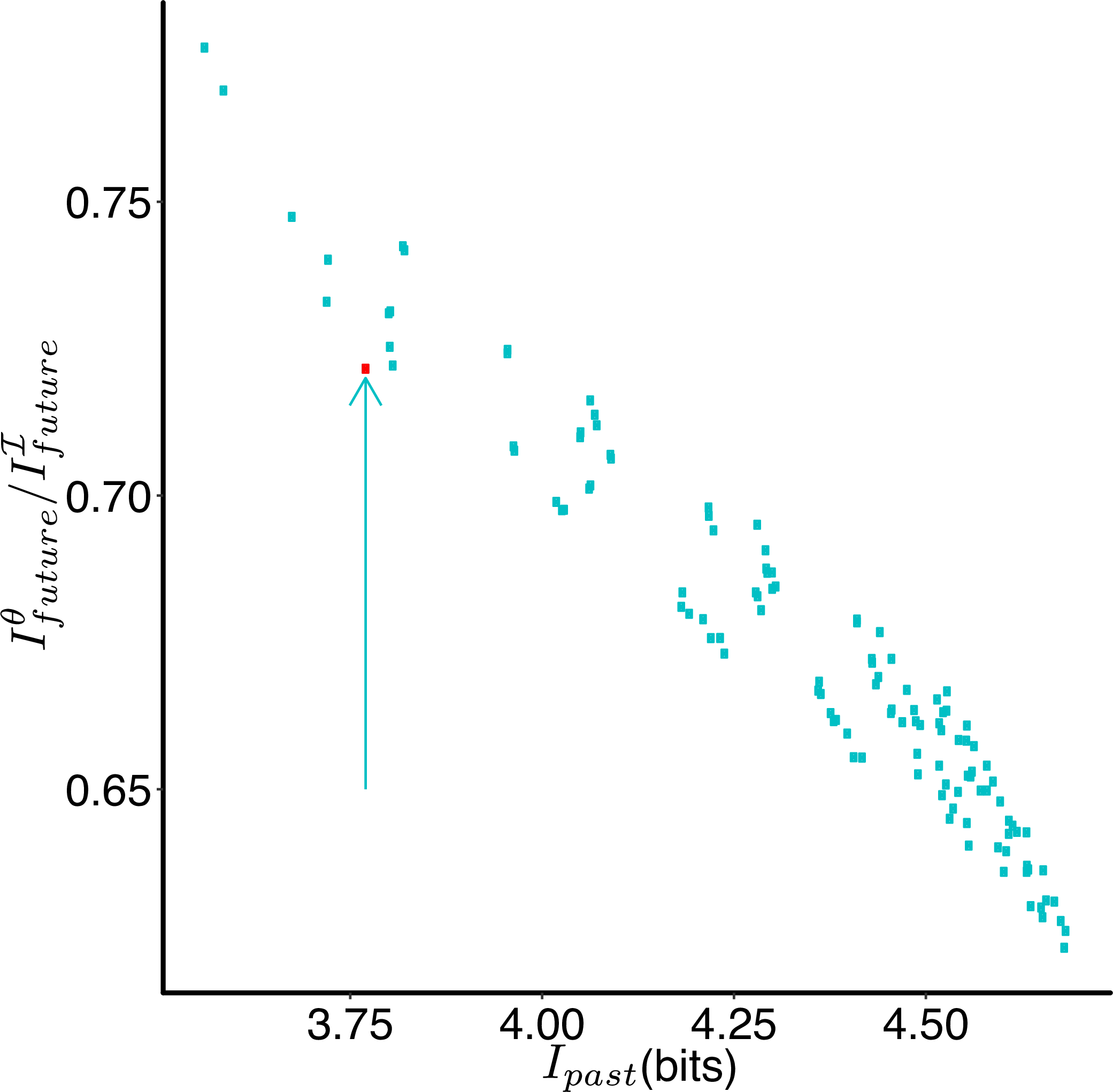

Supplement: S5 Fig — The particular VS 5,6,7 triplet (shown by the red circle and the arrow) that connects with the neck motor center, is one of the most efficient in terms of how much fraction its prediction of its own input is about the future stimulus, while its encoding cost Ipast is modest. (TIF) [file pcbi.1008965.s005.tif]

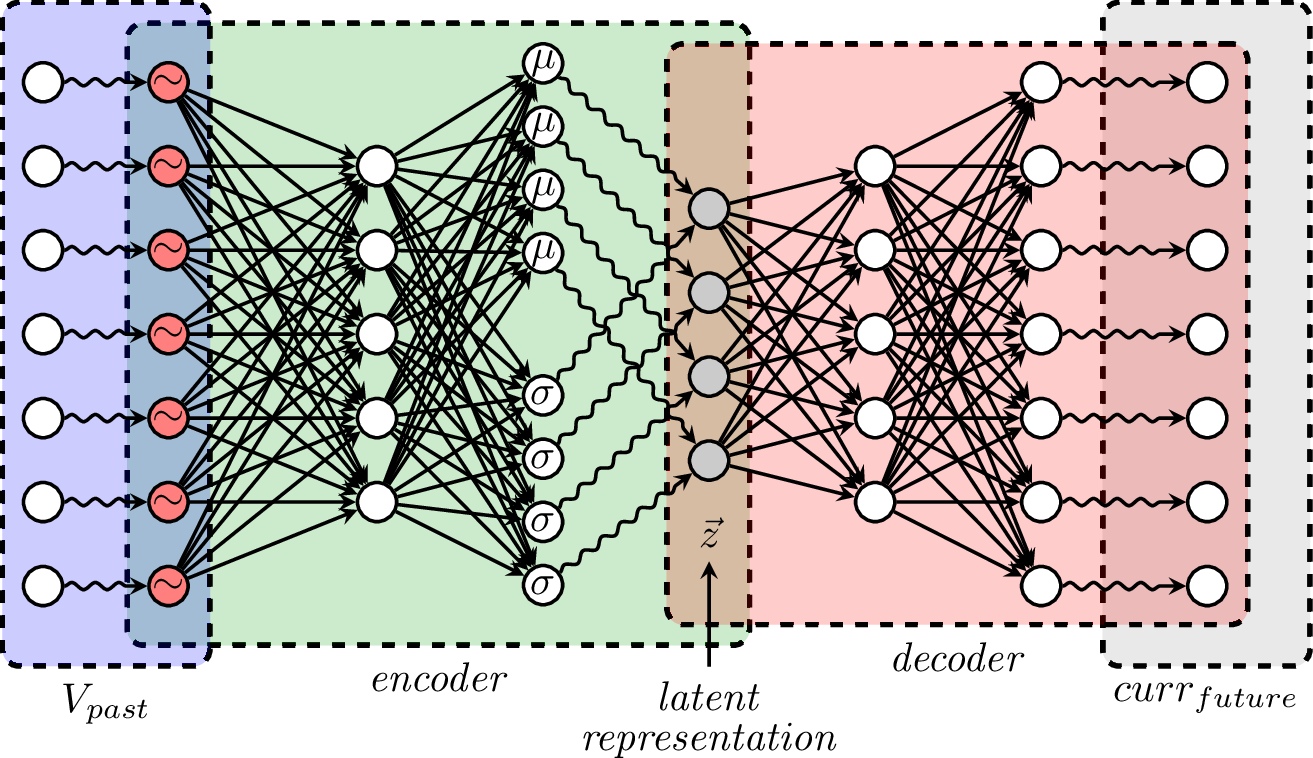

Supplement: S6 Fig — By constructing a variational approximation, the encoder learned a latent representation z→ from the past VS voltages. For training the encoder, we first project the axonal voltages of 20 VS cells to 200 intermediate filters, followed by a batch normalization layer. We then learn a latent representation with z = 2 for easy visualization. Then a decoder of the same structure as the encoder generates samples from z→ and reads them out as the future input current to the VS network. Note the VS network does not have direct access to the stimulus, it uses the correlations between its past and future inputs induced by the stimulus as a proxy for the stimulus correlations, themselves. z→ follows a Gaussian distribution, with parameters as μ and Σ. During training for this VIB, the mean μ and covariance matrix Σ of z→ map the axonal voltages of VS to the future input. When the VIB succeeds, we obtain the predictive representation of the future stimulus by projecting their respective axonal voltages into the latent feature space of z→. (TIF) [file pcbi.1008965.s006.tif]

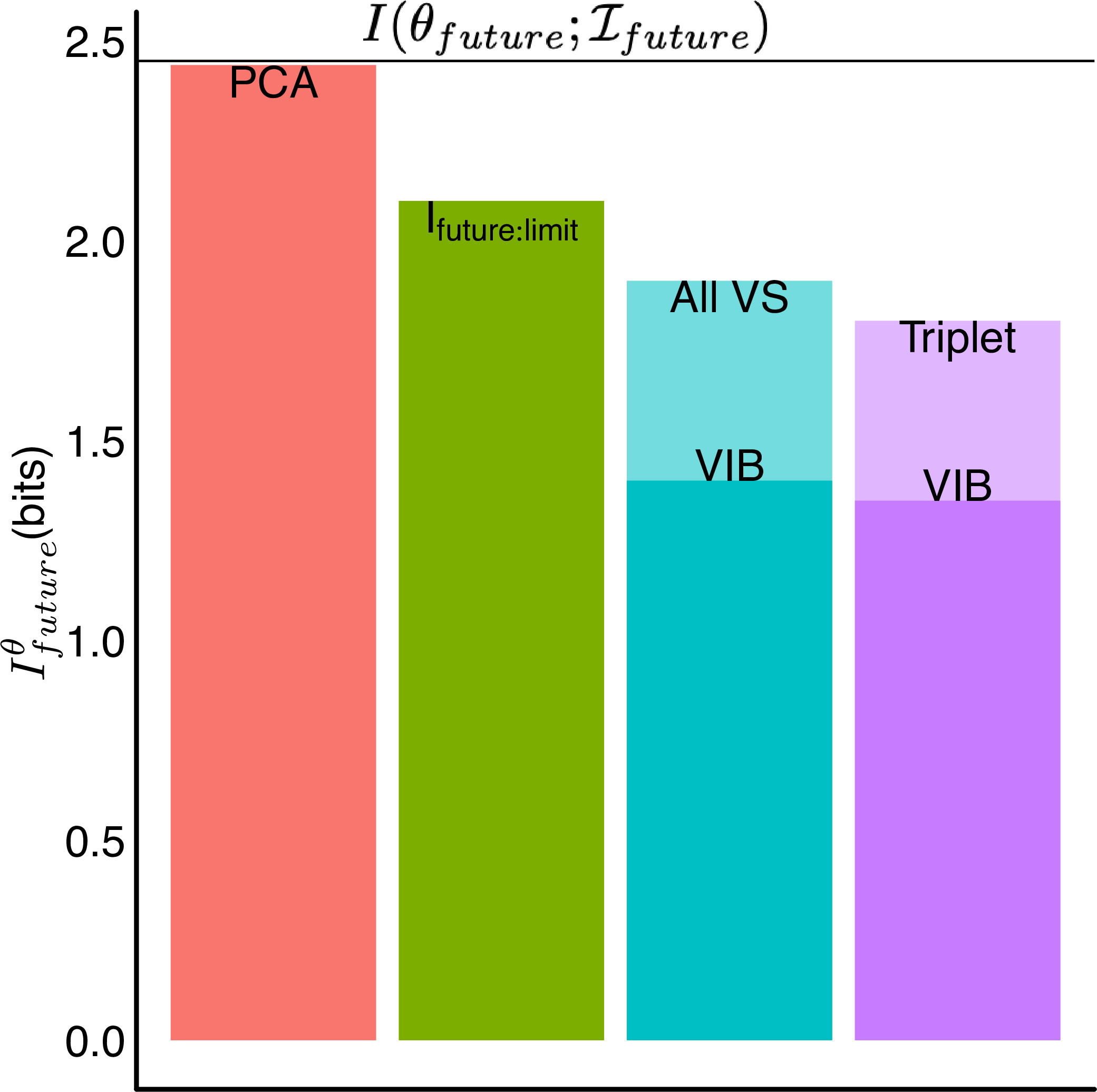

Supplement: S7 Fig — The red bar shows that the PCA projection of the first 2PCs from the input current contains almost all of the stimulus information available at the input current itself. We use this PCA projection to understand whether it is possible to disentangle input stimuli from different quadrants using prediction in Fig 5. The green bar shows the limit on prediction information, based on the information bottleneck method. It corresponds to the point on information curve at the given compression in Fig 4B. The cyan bar corresponds to the predictive information about the future stimulus using outputs from all VS cells. The darker-colored region shows how much information the corresponding VIB captures about the future stimulus. The purple bar is similar to the cyan bar, for predictive encodings of the VS 5-6-7 triplet vs. their respective VIB solution. (TIF) [file pcbi.1008965.s007.tif]

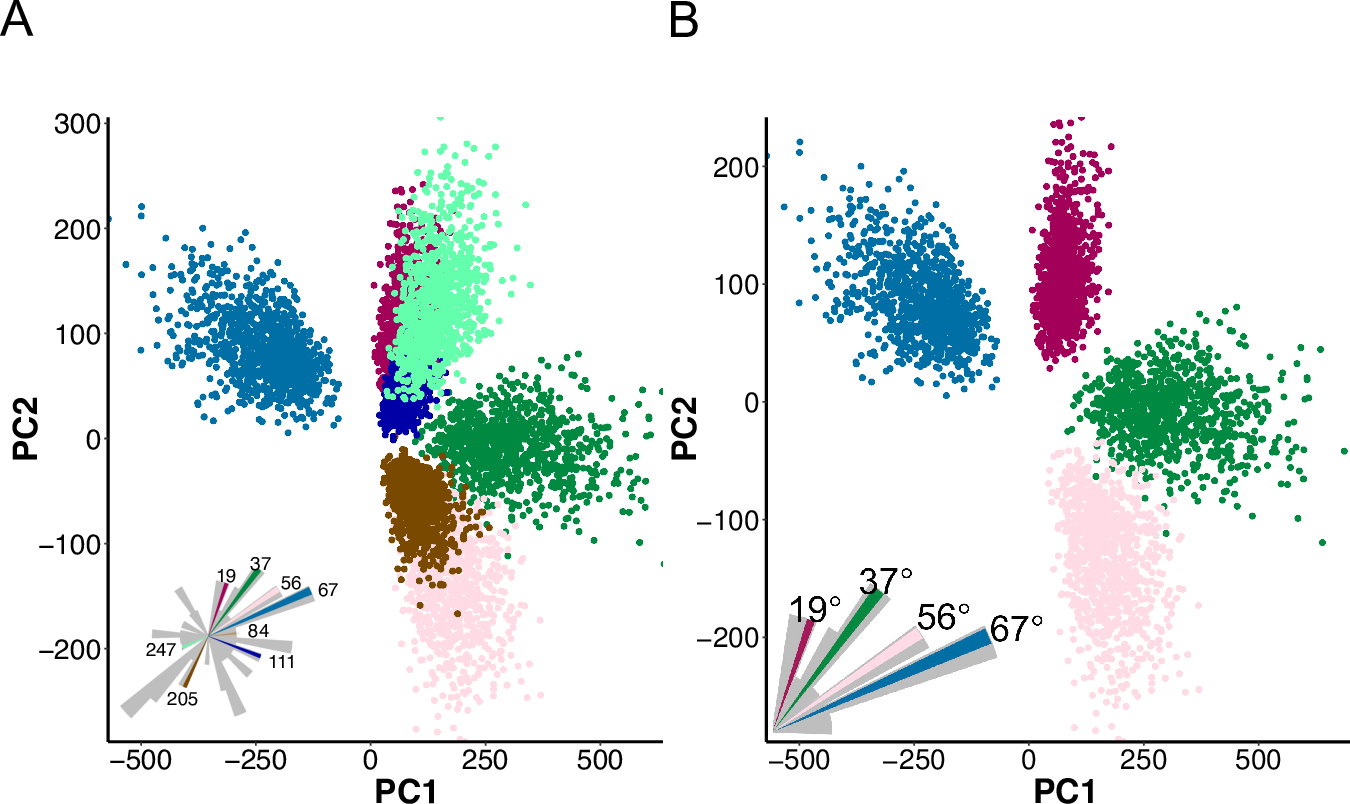

Supplement: S8 Fig — A) The representation of 8 randomly selected stimuli within the plane whose dimensions are the first two principal components of the input currents. Note that there are substantial overlaps between clusters: e.g. the light-green cluster is almost on top of the dark-red/dark-blue clusters. B) The subset of 4 stimuli from A. The only difference, as compared to A, is that all these stimuli have the same pitch/roll directions (clockwise roll and up tilt pitch, i.e. they are all within the 1st quadrant of the fly’s coronal plane). (TIF) [file pcbi.1008965.s008.tif]

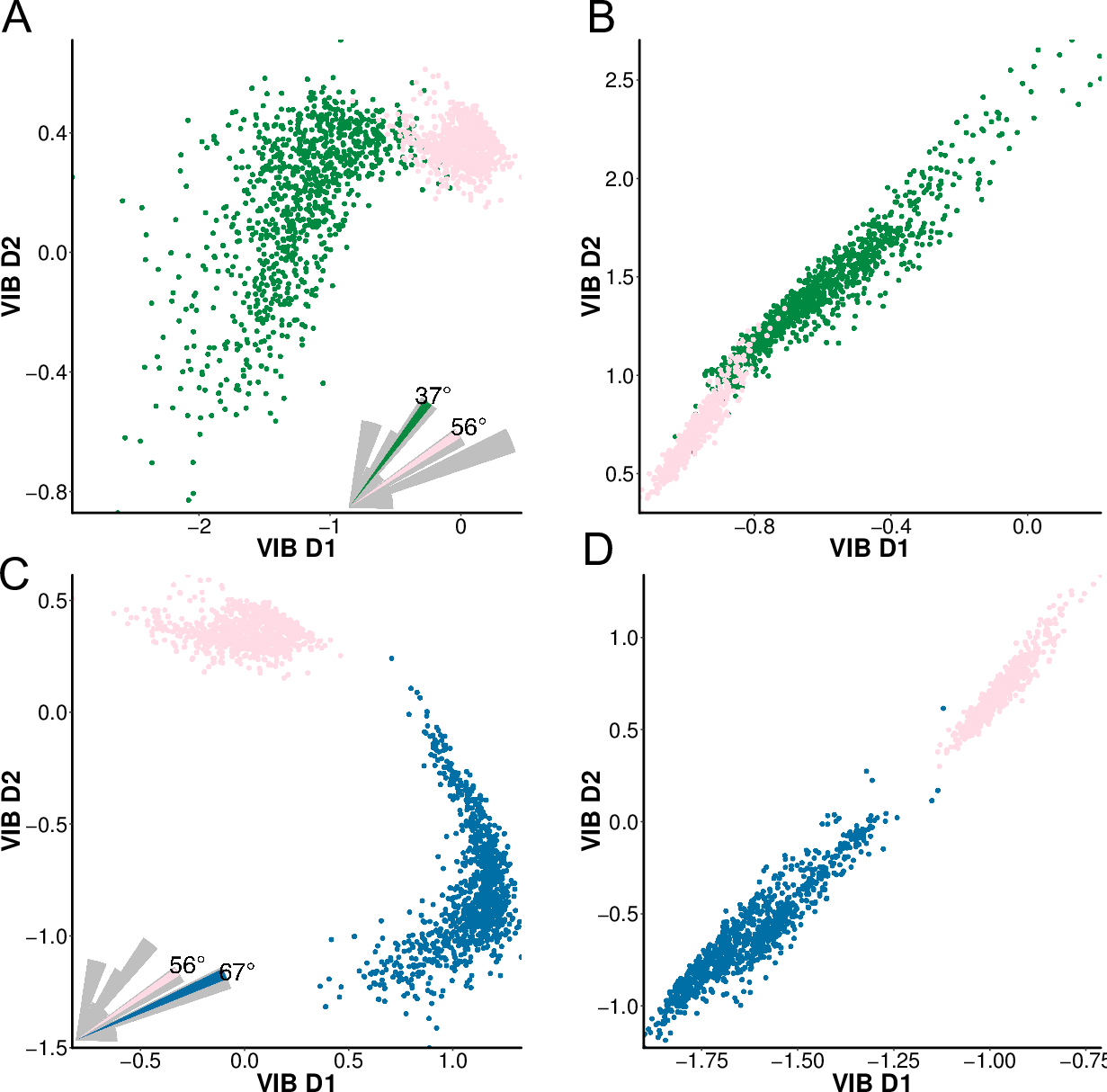

Supplement: S9 Fig — A) The predictive representation of stimuli at 37° and 56° obtained by mapping the respective axonal voltages of the entire VS network to the latent feature space generated by the VIB. B) Similar to A, but using the VS 5-6-7 triplet as input. C) The predictive representation of two stimuli that are much closer in stimulus space: 56° and 67°, respectively. Note that there is no overlap between these two nearby stimuli whereas there is some overlap for stimuli that are farther apart (shown in A). D) Similar to C, but using the VS 5-6-7 triplet as input. (TIF) [file pcbi.1008965.s009.tif]
