# Supplementary material for: Maximally efficient prediction in the early fly visual system may support evasive flight maneuvers
Source: PLoS Comput Biol. 2021 May 20;17(5):e1008965. doi: 10.1371/journal.pcbi.1008965 (PMC8136689; doi:10.1371/journal.pcbi.1008965)
Supplement: Attachment — Submitted filename: Response_letter_Jan_13 (1).pdf [file pcbi.1008965.s010.pdf]

THE UNIVERSITY OF CHICAGO
BIOLOGICAL SCIENCES

STEPHANIE E. PALMER, PH.D.
ASSOCIATE PROFESSOR
DEPARTMENT OF ORGANISMAL BIOLOGY AND ANATOMY
1027 EAST 57TH STREET, ANATOMY 104
CHICAGO, ILLINOIS 60637-1508
PHONE: 773-702-0771 FAX: 773-702-0037

January 13, 2021

We thank both reviewers for their detailed and constructive comments. We have taken these suggestions to heart and have made a serious overhaul to our text and figures. We feel the revisions have significantly improved the manuscript and are grateful for the feedback.

Additionally, we are working to include intermediate data (e.g. output of the simulations as specifically studied here) as well as code to generate the main results of the work. We will be ready to add this to the paper if these revisions are acceptable.

We provided two versions of the revised manuscript: one with highlights of all changes as well as a clean version. We provide detailed responses to each point below.

Sincerely,

Stephanie Palmer, Siwei Wang, Axel Borst, and Idan Segev

Detailed responses to reviewers:

Reviewer #1

1) Line 46: Active control needs more definition. I interpreted it to mean on-going control during the evasive maneuver, rather than the motor activity stemming from a one-time command, and I'm not sure that the subsequent reasoning in this paragraph all supports that interpretation, since a one-time command could create points 1 and 4. = (And control on what timescale? Per wing-beat?) Point 2 seems most relevant, if flies update their escape maneuver in response to continuing changes in the looming stimulus during their maneuver. (Use of "active control" again in line 94).

We thank the reviewer for this comment.
We meant both active and ongoing control, but could have been clearer in the text. "Active" control means that the fly is not eliciting a stereotyped turn when it starts banking, the turn is tailored to the relative position of the fly to the threat and its body position and heading; "ongoing" control refers to the fact that the fly produces active damping to replace the initial rotation with a counter rotation in the latter stage of the evasive maneuver.

We rewrote this part of the manuscript:

These in-flight evasive maneuvers are banked turns, i.e. initiated with a rotation immediately followed by actively damping of that turn to start counter-rotation in the opposite direction. In addition, the maneuvers themselves require active and on-going control: i) The initial rotation is not stereotyped (i.e. purely 90 or on predetermined body axes as are saccadic flight turns [4]), it must be tuned to both the position of the looming threat and the animal's heading at the moment of detection. ii) The subsequent counter-rotation determines how successful the animal is at turning away from the threat by changing its prior flying trajectory. Thus, it also needs to be fine-tuned in an ongoing manner instead of being a simple correction of the initial rotation. iii) Both the initial rotation and the follow-up counter rotation (and their combined trajectories) must be variable even for visual threats appearing on the same position to avoid the predator's trap (e.g. an electric snake can mitigate its prey's predictable, reflexive C-start [5] and thus foil the escape). Previous experiments identified that the mechanosensory circuit, i.e. the halteres, can perform such motor control for voluntary saccadic turns. Thus, it was hypothesized that the halteres are also responsible for the motor control during the escape.

2) Related to the point above: This whole analysis asks how present visual information can be used to predict future visual information, but if it's being used to do this, isn't that a forward model for control, rather than active control? This hinges on the author's definition of "active control", but it seems like 'active control' should be distinct from the kind of control that employs a forward model of what's going to happen. Does the framework used here – in which current signals are informative about future ones -- differ from a forward model?

We agree with the reviewer that we need to clarify the difference between feedforward input from the visual system and active control via the flight control muscles.

We rewrote the introduction to clean this up, particularly this section:

The descending visual inputs useful for haltere control are the global motion responses generated by the large tangential lobula plate cells (LPTC) in the fly visual system. The fly visual system is organized in four largely feedforward layers: retina, lamina, lobula, and lobula plate. Located at the 4th neuropil, these LPTCs have a roughly 30 ms processing lag across many dipterans [15-17]. Because of this processing lag, it is unlikely that evasive maneuvers can access visual information through feedback during the escape. Instead, we propose that the visual information of ego-motion at the time of threat detection may play a new feedforward role. Said another way, by using its own past visual input before the evasive maneuver to predict the its future visual experience during that maneuver, a fly bypasses this processing lag and still uses visual inputs for active control. This so-called "bottom-up" prediction exploits the temporal correlations between past and future visual stimuli (due to the stereotypy in the escape behavior) during evasive maneuvers. Such bottom-up prediction exists in the vertebrate retina [18, 19], and ensures fluid interaction with the external environment. It was also shown to be important in the formation of long-term memory [20]. In our case, the escape trajectory depends on the threat angle relative to the fly's heading [21]. Where and how the escape maneuver begins constrains how it will unfold, giving the visual system ample predictive power with which to feed forward into active flight control. We show how this bottom-up prediction provides information about the future sensory input, subverting delays in the visual input stream. Because blowflies are known to execute higher velocity saccadic turns than Drosophila, this bottom-up prediction will be even more important for blowflies' shorter and faster evasive maneuvers.

3) The authors note that these predictions are only possible because of correlations (not necessarily low-order) in the trajectory during these evasive maneuvers. A vanilla explanation of these results would

be that simple, second-order trajectory correlations account for a good fraction of trajectory variance, and the VS network predicts the future well because it's a good representation of the present. Can one ask how much variance in future state is explained by current trajectory? I guess I'm asking about whether some of this analysis can also be done using simple representations of temporal co-variance, and how different the results are when more sophisticated, information theoretic methods are used.

We agree with the reviewer that this is an important point.

We have added a supplementary figure to show that linear correlation between the past and the future egorotation can only explain a small fraction of the total variance, meaning that the higher-order correlations that are encoded by the VS system and quantified by our information theoretic analysis push beyond this kind of linear relationship. That figure is included here:

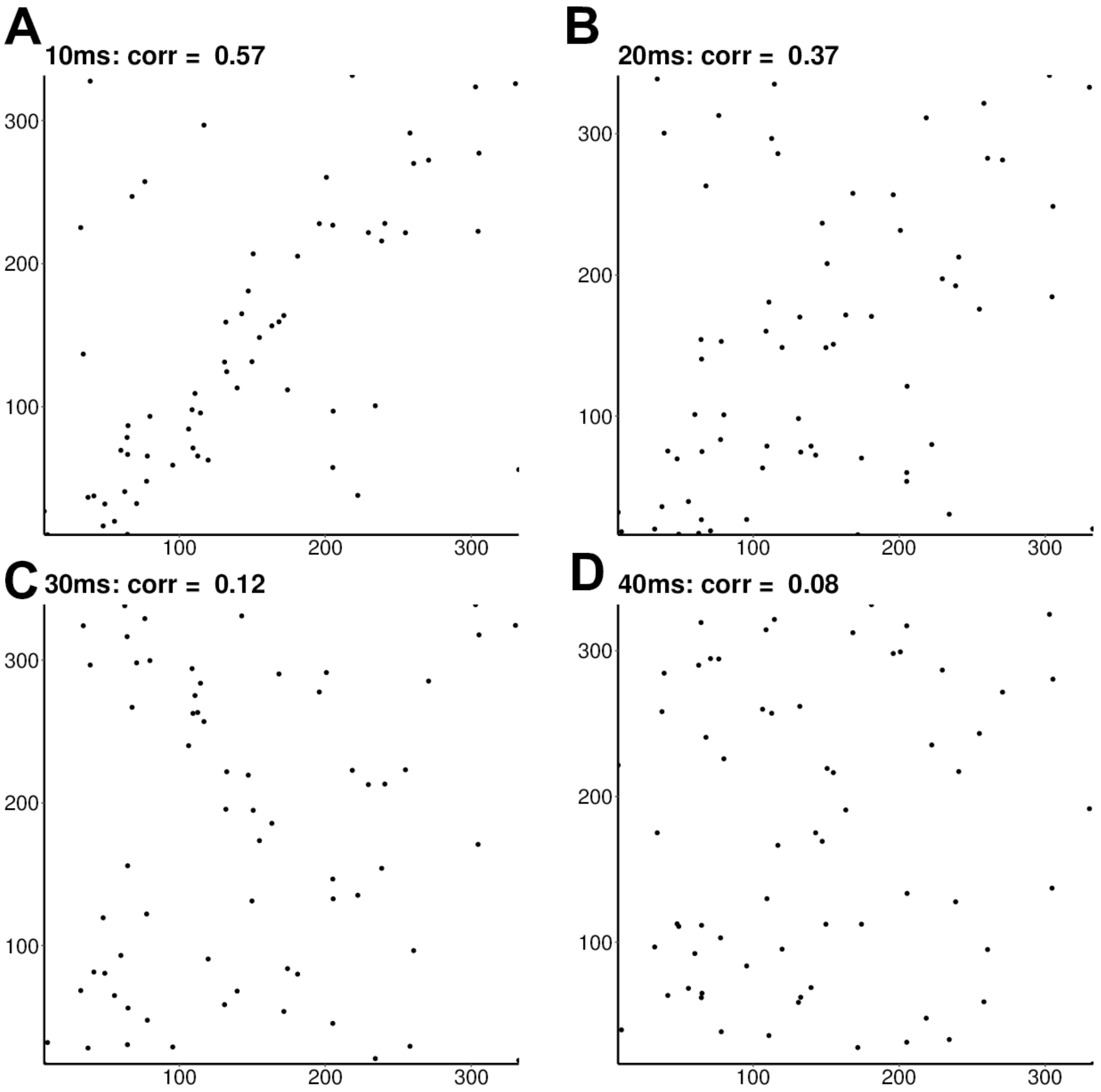

New SI Figure 3: Linear correlation between the past egoroation and the future egorotation at different time lags in the evasive maneuver}. A) The correlation between the egorotation distribution at 10ms before the onset of evasive maneuvers and the egorotation distribution 10ms into evasive maneuvers. B) Similar to A), but for the egorotation distribution 20ms into evasive

maneuvers. C) Similar to A), the egorotation distribution 30ms into evasive maneuvers. D) Similar to A), the egorotation distribution 40ms into evasive maneuvers.

4) Input currents to VS cells are critical to define well, given their importance in this study: they were nowhere defined that I could see, though might be in a referenced paper. For instance, I expect these currents to include excitatory and inhibitory inputs from the LMD model, but do they allow those conductances to shunt current? For instance, if both excitatory and inhibitory conductances are high, is the current their sum, or an appropriately weighted sum reflecting the VS membrane voltage? Does it make a difference?

We thank the reviewer for this comment:

In the revised materials and methods, we have added a new paragraph explaining more carefully how the input current is computed:

The final step before simulating the VS network's response is to simulate its upstream local motion detection at the fly's retina. We use 5000 local motion detectors (LMD) evenly distributed on the unit sphere. Each LMD contains two subunits that differ by 2° in elevation. Their responses are $R(t) = (f * V_1)(t)(g * V_2)(t) * (g * V_1)(t)(f * V_2)(t)$ *where* $V_{1,2}$ *are photoreceptor responses at two neighboring locations.* f *is the low pass filter and* g *is the high pass filter [96]. Each VS dendrite takes as input the output of the LMDs that fall into its respective field. The receptive field (RF) of these dendritic compartments are modeled as 2-D Gaussian* $S(x,y) = \frac{1}{2\pi\sigma_x\sigma_y} exp(-[\frac{(x-x_{ctr})^2}{2\sigma_x^2} + \frac{y^2}{2\sigma_y^2}])$ *with azimuth* σ_x *= 15° and elevation* σ_y *= 60°, tiling along the anterior-posterior axis (e.g. the centers of these receptive fields are located at 10°, 26°, ..., 154° for VS1-10 respectively, see detailed configuration in [39]). The input current of an individual VS dendrite is the weighted sum of the synaptic conductance load from both excitatory and inhibitory inputs multiplied by the potential, i.e., with the excitatory conductance load as* $g_E = \sum_{x=-180^\circ}^{180^\circ} \sum_{y=-90^\circ}^{90^\circ} S(x,y)[R(t)(x,y)]$ *, the input current is then* $g_E * E_E + g_I * E_I$ *(*$E_{E\ or\ I}$ *are reverse potentials relative to excitatory/inhibitory resting potentials), it is kept between -2.5nA to 2.5nA to be realistic [48]).*

5) Related to the point above: how much do these results depend on the timescale of filtering of the local motion detectors? To obtain temporal frequency tuning of ~1 Hz (as in typical VS recordings), the delay line in any motion detector must do some substantial filtering over time, say 150 ms, which would cause reasonably long autocorrelations in the velocity signals. How does this affect the ability of this network to encode predicted changes in the future signal on timescales of the 40 ms maneuver? I can't quite see how a long time delay in the motion detector could work with these short, fast maneuvers.

We thank the reviewer for this comment.

Although motion detection in the VS network uses long low-pass time constants of 150-200ms to achieve its optimum temporal frequency in the steady state, short abrupt maneuvers will also elicit transient detector responses. This was first shown in experiment [Reisenman C, Haag J, Borst A (2003) Adaptation of response transients in fly motion vision. I: Experiments. Vis Res 43: 1291-1307.] and validated by modeling. Namely, a single VS cell emits transient responses at the timescale of milliseconds. In addition, previous theoretical work \cite{trousdale2016, wang2017efficient} showed that

the population coding scheme in the VS network efficiently encodes constant speed rotational motion using only this transient response, i.e., the output of the initial 10ms after encountering a motion stimulus.

To clarify this, we took a few steps:

i) We rewrote the introduction to clarify that we focus on transient responses of the VS network at 10ms scale:

Although both the local motion detection at the retina \cite{Creamer2018, Haag2004pnas} or the global motion processing at the VS network have long low-pass time constants of 150-200ms to achieve its optimum temporal frequency, these considerations are specific in the steady state. Previous experiments \cite{Reisenman2003} and modeling \cite{borst2003} showed that single VS cells elicit transient detector responses for short abrupt maneuvers at the timescale of milliseconds. This makes the VS network suitable for evasive maneuvers.

ii) We changed the Material and Methods to emphasize that the axonal voltage in all of our subsequent analyses is the transient temporal average. Because the maximum firing rate of the descending neuron connecting to the neck motor center is 100 Hz \cite{weber2008eigenanalysis}, we use the transient temporal average of the resulting axonal voltage $\overline{V}^{tr}_{past} = 1/T\int V_{past}(t)dt$ (in the main text, the $\overline{V}_{past}$ are temporal average. For simplicity, we use V_{past} instead).

Minor points:

1) Line 87: Not sure how an active counter-rotation differs from a counter-rotation.

They are the same in terms of the resultant trajectories, but we add “active” to emphasize what kind of control is required.

Active counter-rotation is a two-phase maneuver: it first actively damps a previous rotation, meanwhile initiating another rotation in the opposite direction. The banked turn includes a rotation followed by a counter-rotation which must be actively controlled (changing direction from the initial rotation). Said another way, passive damping can only asymptotically reduce roll and pitch rates and not reverse them, a banked turn thus requires active production of counter-torque.

We use the following sentence in the introduction to clarify this (line 59-61):

These in-flight evasive maneuvers are banked turns, i.e. initiated with a rotation immediately followed by actively damping of that turn to start counter-rotation in the opposite direction.

2) Line 146: Citation for TF-tuning of local motion detectors only references theory for and recordings from an LPTC, which has spatially integrated, opponent-subtracted signals from local motion detectors. Creamer et al. 2018 showed that individual local motion detectors in Drosophila also have this tuning (before opponent subtraction).

We thank the reviewer for this comment, we cited Creamer et al. 2018 in the following sentence (line 132-134):

Although both the local motion detection at the retina \cite{Creamer2018, Haag2004pnas} or the global motion processing at the VS network have long low-pass time constants of 150-200ms to achieve its optimum temporal frequency, these considerations are specific in the steady state.

3) Line 154: Add citations for LPLC2 and LC2. I don't think the authors mean LC2 – it should be LC4. This will be Klapoetke et al. and Ache et al.

We thank the reviewer for pointing out this error, we corrected it in line 308 to mention "LPLC2 (Klapoetke et al. 2017) and LC4 (Ache et al. 2019)" with proper citations.

4) Fig 1C: Mercator projection seems to only show 180 degrees of azimuth. Perhaps scales would be helpful if a full 360 is not being shown?

We corrected Fig 1C to show that this projection only shows 180 degree of azimuth.

C

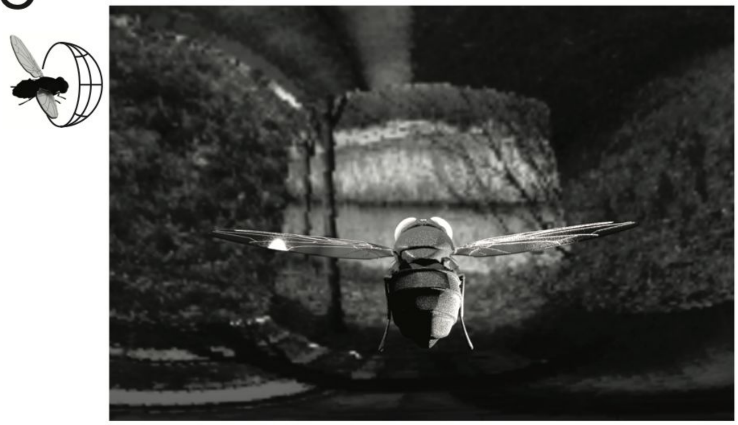

5) Line 301: Talking about contrast heterogeneity. Not necessarily required for this model, but might be important to mention recent work in Drosophila on contrast normalization in the LMDs preceding LPTCs (Drews et al. and Matulis et al.). These sorts of effects almost certainly also exist in bigger Diptera.

We added both Drews et al. and Matulis et al. as citations for contrast normalization (line 285-287).

It was identified that such contrast normalization denoise the dendritic inputs to the VS network \cite{Mauss2015}, recent work further showed that this contrast normalization acts nearly instantly \cite{Drews2020}, compatible for the processing during evasive maneuvers.

6) Is the result in Figure 2 just due to averaging over space? Figure S3 seems to argue strongly against that, and the authors might consider moving that to a main figure.

We moved Fig S3 to the Fig2 and modified the respective results section. We also would like to clarify that Fig 2 and Fig S3 are different: Fig 2 focuses on showing how predictive information encoding is different when gap junctions are at different locations. Fig S3 shows the difference between the encoding of predictive information and encoding instantaneous input efficiently. The functional role of axonal gap

junction is more similar to a kind of linear interpolation, as shown in [Cuntz et al. 2007]. It produces a robust representation of rotational optic flow.

We added the following sentence to clarify the result this point:

The upstream input from local motion detectors brings much more substantial synaptic conductance load on the dendrites than the dendritic-axonal leak, so placing a gap junction at the dendrites has negligible effects on the VS output [70]. However, placing gap junctions between the axonal compartments performs a kind of linear interpolation along the lateral (azimuth) direction. This removes corruption of the ego-rotation signal arising from the inhomogeneous contrast distribution of the natural visual scene (and not from the global ego-rotation signal, this is analogous to how dendritic integration at single VS cell removes corruptions along elevation, shown in [71]).

7) Figure 3: Is it worth showing all triplets, rather than the cross bar? Would give a better sense of how many of them lie close to or far from the limit. Same with Figure 4B.

It is a little challenging to show a scatterplot with the same scale and retain readability of the data. Instead, we have included separately scaled scatterplots as part of the Supporting Information.

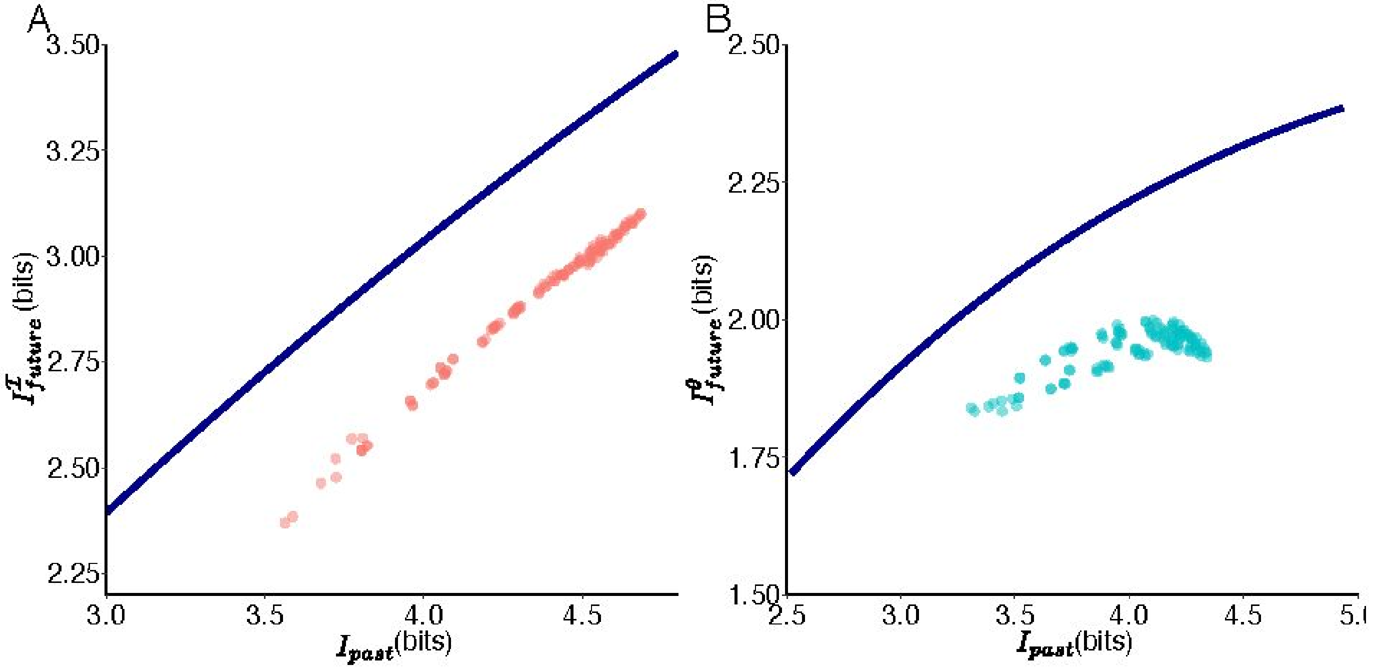

8) Since this is all information theoretic, is there a proposed method for the read out of the future information from the current state? Or just that it's there?

We thank the reviewer for asking this important open question. We changed the discussion to clarify why we do not directly suggest a readout mechanism:

How the fly brain reads out the predictive information from the VS system is an important open question. Although the information contained by the temporally averaged output of the VS population response can be read out linearly, this may not be how readout takes place in the real downstream pathways of the VS network. For example, there are two descending DNOVS neurons (DNOVS1 and DNOVS2) connecting to the output from the triplet VS 5-6-7 [66, 72, 87, 88]. While DNOVS1 is a graded neuron, the DNOVS2 is a spiking neuron (with firing rates up to 100Hz). DNOVS2 introduces a substantial nonlinearity such that the biological readout may not be a simple integrator. In addition, the output of the VS network may be further combined with outputs from other LPTCs before they reach a motor control center [39].

9) Figure 5: numbers representing the angles are pretty unreadable. Please enlarge.

We changed Figure 5 (as well as supplementary Fig 8 and supplementary Fig 9) to a larger font size.

10) Figure 5BD: I don’t understand why these two VIB dimensions are so highly correlated. Does this mean a single dimension could do the VIB encoding in the case of this triplet?

No, we agree that the overall correlation is high, but this does not indicate that a single dimension can do encoding as well as the 2D VIB, or the VS triplet.

Reason 1: VIB only captures 70% of the predictive information compared to how much is encoded in the VS triplet. We use the VIB as a proxy to make an educated guess about the functional role of predictive information encoded by the VS triplet because a) we cannot easily show a 6 dimension distribution. b) Any computations possible by encoding 70% of the predictive information will definitely be implemented at higher performance levels by the actual VS triplet.

Reason 2: The 1st dimension encodes 1 bits about the egorotation, the 2nd dimension encodes additional 0.3 bits about the egorotation. This shows they encode different egorotation-relevant features.

Reason 3: The correlation coefficients for each individual egoroation are different, this makes the discrimination between two different egorotations better using two dimensions. Using the clusters of 37 and 56 degrees as examples, their overlap in the 2D space is much less compared to using either D1 or D2 only (e.g., using “VIB D1” as an example, the 37 degree cluster occupies [-1,0.2] and the 56 degree cluster occupies [-1.13, -0.4], merely using “VIB D1”, their overlap covers more than 87% of the 56 degree cluster and 50% of the 37 degree cluster. By contrary, the 2D area overlap between these two clusters is much smaller: by comparing the data ellipses drawn at 99% confidence level. We observe

that the overlap reduces to 16% for the 56 degree cluster and 2% for the 37 degree cluster, respectively.

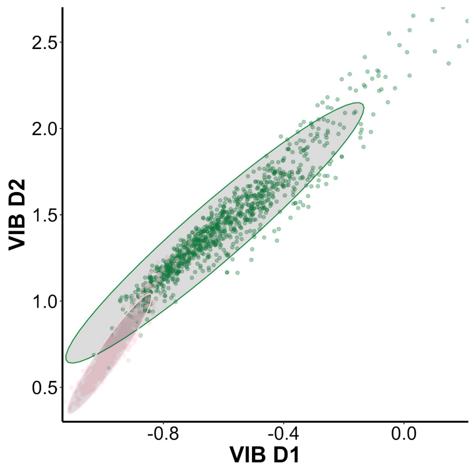

This is also a geometric interpretation of reason 2 that combining 2D VIB encodes more information about egorotation.

Reason 4: We used a vanilla lossy variational autoencoder in this VIB latent representation without specifically controlling the structure of the latent representation. This is beyond the scope of current work. It is an active research subfield in machine learning (https://openreview.net/forum?id=r1gxEILFu4, https://arxiv.org/abs/1610.03454). We definitely hope future work can use biologically plausible constraints on this latent representation.

11) Line 589: capitalize Drosophila

We corrected this *Drosophila* and double checked that all "*Drosophila*" are capitalized and italicized.

12) Line 634: need equation for local motion detection.

We thank the reviewer for this comment.

We added the equation for local motion detection "$R(t) = (f * V_1)(t)(g * V_2)(t) * (g * V_1)(t)(f * V_2)(t)$ where $V_{1,2}$ are photoreceptor responses at two neighboring locations. f is the low pass filter and g is the high pass filter" to the materials and methods

13) Line 638: reference V_past on both sides of equation, T is undefined. (minor, change the left hand side w/ the mean sign)

We corrected the left hand side to show that it is the temporal average of the right hand side. We also put the following clarification alongside with it "we use the transient temporal average of the resulting axonal voltage $\overline{V}^{tr}_{past} = 1/T \int V_{past}(t)dt$ (in the main text, the $\overline{V}_{past}$ are temporal average. For simplicity, we use V_{past} instead). "

14) Line 667: variable k appears undefined. Some capitalizations required in this paragraph.

We thank the reviewer for this comment. We corrected all K-nearest neighbors using the capital K.

15) Fig S2: Only 2 labeled panels, but don't match the figure caption.

We thank the reviewer for the comment, we corrected the Fig S2 with subfigure labels (A-E) and corrected the figure caption to be consistent.

Reviewer #2:

1 - In reality evasive maneuvers are triggered by a specific object in the visual field (e.g. an obstacle or a predator). Naturalistics scenes generated by the authors do not have, however such visual obstacles matched to the behavior - they are just images from the van Hateren database. That is the statistical structure of the scene and the evasive behavior are independent of each other. How does this affect the interpretation of the results? Could it be that after matching visual scene content to the behavior the ratio of I(theta, V) / S(theta) (Fig. 1B) would be much closer to 1? I think this point is central to the argument of the paper and should be explicitly discussed (and/or supported with additional analysis).

We thank the reviewer for this comment. We agree that we need to clarify this important issue.

In the original paper describing the escape trajectories of Drosophila, they used three different expansion velocities (0.8°, 1.6°, 2.1°/ms). The sensory motor delay they reported are 84±17ms, 75±10ms and 43±8ms. They concluded that while the expansion speed definitely changes the sensory-motor delay (faster speed results in shorter sensory-motor delay), the response dynamics were identical when they temporally aligned all trajectories to the same acceleration threshold. They also tested three different maximum stimulus expansion angles (32°, 64°, 165°) in combination with two post expansion conditions (all lights on, a white background, or having the dark stimulus remain constant). They did not observe any significant differences in terms of escape trajectories in all six conditions.

Because of the above observation, their published dataset does not include the expansion conditions, i.e. the speed or size at each time point, for the respective escape trajectory. The published dataset is temporally aligned at the start of evasive maneuvers: by only including -40 to 40ms around this time point, it is impossible to know when these threats appear, and what are their sizes at the start of evasive maneuvers. In addition, they did not provide the respective sensory-motor delay specific for each trajectory. Lack of such essential information prevents us from realistically simulating a visual background with an appropriate visual threat.

Compared to the natural scene background, a visual threat as large as (43+8)*2.1=107° in diameter is similar to a big piece of cloud, water or other larger, lower contrast objects in terms of their respective local motion responses. Therefore we hypothesize that using the natural scene background won't change the result significantly.

It is also possible that the ensemble of visual threat used in the original experiment [Muijres et al. 2014] is not sufficient to show how the fly may change its course of escape trajectories with respect to different expansion size/speed. For example, The size of visual threats range between (84-17)*0.65 = 43.55° to (43+8)*2.1=107° in diameter. This is merely a two-fold difference. We believe that realistically testing if expansion speed (up to a broader range) changes the escape trajectory is an interesting future direction.

2 The authors highlight the importance of the gap junctions (GJ) as a key biophysical component necessary to encode the predictive information (e.g. Fig. 2). These gap-junctions are a subset of parameters of the VS model. How many other parameters does the model have? Are there other parameters which might dramatically affect the network performance other than GJs? In other words - what makes GJs a unique subset of parameters from the perspective of predictive information coding?

We thank the reviewer for this comment.

Our experiment collaborator (Borst) had identified all the single cell parameters by corresponding single cell current injection experiment and compartment model fitting.They identified R_m, R_a, C_m, g_{exc}, g_{inh} for each VS cell. The gap junctions between the axons of VS cells are the only connectivity that gives the VS network a chain-like architecture. Mechanistically such connection makes the VS network denoise its axonal voltages in the horizontal direction, different from the vertical direction denoising takes place at the dendritic branches of each individual VS cells [Borst & Single 1998]. Moreover, it implements the electrotonic segregation between the dendrites and axons: such segregation makes the axons have broader receptive fields compared to the dendrites and enable them to output more robust population coding [2008 nat neuro yishai paper]. Therefore, it is a dominant factor for network performance.

We added the following paragraph to the result section to justify why we focus on the functional role of axonal gap junctions.

Previous work identified the R_m, R_a, C_m, g_{exc}, g_{inh} for each VS cell [48] and observed that the network performance of the VS network is dominated by its chain-like wiring architecture [37, 48-50]. Each VS cell only connects with other VS cells with immediately neighboring receptive fields, e.g. VS2 only connects to VS1 and VS3. Meanwhile, the VS1 and VS10 cells show reciprocal inhibition [69]. Previous dual-recording experiments [49] have shown that VS cells connect amongst each other through electrical synapses. Further dye-coupling experiments also have shown that these electrical synapses were gap junctions [50]. In [37], they identified that these gap junctions are located at the axons of VS cells. Anatomically, these gap junctions are the only connection between the VS cells. Mechanistically, the VS network implements an electrotonic segregation through these gap junctions: all VS cells show broadened receptive fields at their axons compared to those at their dendrites. These broadened receptive fields improve the robustness of visual motion encoding [37, 70] at the output of the VS network. The upstream input from local motion detectors brings much more substantial synaptic conductance load on the dendrites than the dendritic-axonal leak, so placing a gap junction at the dendrites has negligible effects on the VS output [70]. However, placing gap junctions between the axonal compartments performs a kind of denoising along the lateral (azimuth) direction by removing corruption of the ego-rotation signal arising from the correlated structure of the natural visual scene (and not from the global ego-rotation signal, this is analogous to how dendritic integration at a single VS cell removes corruptions along elevation, shown in [71]). Gap junctions also support a robust [37] and efficient [44] subpopulation readout scheme: the output from the VS network arises from subpopulations of adjacent cell triplets, which target different downstream areas [39, 72]. In particular, the VS network connects to the downstream descending motor neurons or neck motor neurons only through the VS 5-6-7 triplet of cells [48, 72], which have dendritic receptive fields located at the center of the fly's field of view. Therefore, these axonal gap junctions are dominant for network performance.Here, we investigate i) if axonal gap junctions improve prediction; ii) how such improvement is different from efficient coding of instantaneous input previously shown in [44].

3 - The idea of encoding the information about the organisms own future behavior is interesting, but could be perhaps better discussed. From a more "control-theoretic" perspective, the aim would be to encode the stimulus, incorporate it into the model of the environment, and then generate action which maximizes probability of the obstacle avoidance, given the flies current belief (or prediction) about the environment. Can one think of encoding the predictive information as extracting bits relevant only for such "control-theoretic" planning? I think it could be better explained and positioned in the context of the current literature.

We thank the reviewer this comment: we add the following paragraph to discussion about how prediction *fits in a control-theoretic perspective:*

Previous work had proposed that the halteres may implement a proportion-integration (PI) controller framework to generate motor commands [57]. In this framework, we hypothesize that feedforward visual control may have a functional role similar to the integration term: both are meant to reduce noise for high frequency signals, i.e. high velocity rotation in the evasive maneuver. A key difference between visual prediction and the integral term is that visual prediction does not merely respond to past errors, it gives an estimate of the future: it supports fine-scale discrimination of future ego-rotations at high velocity. This noise reduction is better suited for the abrupt, brief nature of evasive maneuvers, whose past error (before the initiation of evasive flight) may not be useful. Recent experiments [11] identified that visual information can shift the dynamic range of the motor neurons in the halteres. Namely, the halteres themselves obtain proportional gain for ego-rotation up to 2500° /s from its own mechonsensory input. The visual prediction then induces a shift in the haltere dynamic range so that they can output appropriate motor commands if a future ego-rotation is beyond 2500° /s and up to 5300° /s. Whether this linear PI controller is indeed implemented by the halteres is an interesting direction for future research.

4 - If I understood correctly, at best, predictive information is only around 50% of the entropy of the body rotation (Fig. 1B). If it is so - then to avoid the obstacle the flies still needs a lot of information. Where does it come from? How can partial information be used to perform the maneuver much earlier? It would be good to discuss these aspects explicitly.

We agree with the reviewer. There are many other neural circuits that can provide input to the halteres to general corresponding motor commands (i.e., the control loop hypothesis), or they generate motor commands themselves. The investigation of this multi-sensory integration is beyond the scope of this study (we focus on visual prediction). However, it is an exciting future direction that merits both experiment and theory investigations.

We add the following citations to show that we are aware of the multi-sensory integration paradigm:

Fig.1B shows that the predictive information $I_{future}(\theta, \Delta t)$ in the VS output voltages captures nearly 50% of the entropy of the future escape trajectory. This is because where and how the escape maneuver begins constrains how it will unfold, giving the visual system ample predictive power. This suggests that the predictive information encoded by the VS network is an important information source for evasive flight behaviors in the natural environment. This also supports that the haltere is a multisensory integration circuit, other sensory modalities, e.g. antenna [58, 59], ocelli [60{62] or wing reflexes [63, 64] may provide additional motion information.

Antenna:

Hengstenberg R. Gaze control in the blowfly Calliphora: a multisensory, two-stage integration process. Seminars in Neuroscience. 1991;3(1):19{29. doi:10.1016/1044-5765(91)90063-t.
Gopfert MC, Robert D. The mechanical basis of Drosophila audition. The Journal of experimental biology. 2002;205:1199{1208.

Ocelli:

Parsons MM, Krapp HG, Laughlin SB. A motion-sensitive neurone responds to signals from the two visual systems of the blowfly, the compound eyes and ocelli. Journal of Experimental Biology. 2006;209(22):4464-4474.
Budick SA, Reiser MB, Dickinson MH. The role of visual and mechanosensory cues in structuring forward flight in Drosophila melanogaster. Journal of Experimental Biology. 2007;210(23):4092{4103. doi:10.1242/jeb.006502.
Krapp HG. Ocelli. Current Biology. 2009;19(11):R435-R437. doi:10.1016/j.cub.2009.03.034.

Wing flexes

Sane SP, Dieudonne A, Willis MA, Daniel TL. Antennal Mechanosensors Mediate Flight Control in Moths. Science. 2007;315(5813):863{866}. doi:10.1126/science.1133598.
Eberle AL, Dickerson BH, Reinhall PG, Daniel TL. A new twist on gyroscopic sensing: body rotations lead to torsion in flapping, flexing insect wings. Journal of the Royal Society, Interface. 2015;12:20141088. doi:10.1098/rsif.2014.1088.

5 - The authors should dedicate more space to explain the relationship of this work to their previous paper [57]. In particular - if 57 claims that almost entire information about the rotation theta_t is encoded in the VS network state - how substantial is the current advancement? After all, efficient coding and coding of predictive information will start to diverge when the bottleneck is strong (i.e. the network can retain only a small proportion of bits from the input). Even if my understanding of [57] is incorrect, this should be much more explicitly discussed.

We thank the reviewer for this comment.

The one key factor lacking in [57] is that we did not consider in that work whether the encoding is behaviorally relevant.

We changed our introduction (line 137-152) to emphasize this:

In addition, recent theoretical work [45, 46] showed that the population coding scheme in the VS network efficiently encodes constant speed rotational motion using transient response only, i.e. the output of the initial 10ms after encountering a motion stimulus. This effect was dependent on the positioning of gap junctions between the axons of neighboring VS cells, and has been experimentally validated in [37, 47]. This work suggests that the VS network can encode motion information on a timescale compatible with evasive maneuvers. However, without determining if the output of the encoding scheme is behaviorally relevant, this cannot determine if such processing is useful for downstream motor control. In naturalistic behaviors, ego-motion is highly dynamic. Encoding a past ego-motion is only useful if it is informative for the animal's future experience. Merely maximizing information about the instantaneous motion is not the challenge the animal is facing during evasive maneuvers; the VS network must extract the predictive information about future

motion. In order to identify whether the specific wiring architecture of the VS network supports evasive maneuvers, we ask whether it encodes predictive information about future ego-motion using within its own transient responses.

We also changed the Results section II to explain the difference between these two encoding schemes, especially highlighting how much they rely on having the chain-like architecture in the VS network:

We also observe that the presence of axonal gap junctions improves the encoding of predictive information much more than it improves the encoding of the instantaneous ego-motion [46] (Fig.2B). Comparing $I_{future}(\Delta t)$ *with* $I(\theta_t; V_t)$*, we observe that the presence of axonal gap junctions improves* $I(\theta_{t+\Delta t}; V_t)$ *and* $I(I_{t+\Delta t}; V_t)$ *about 100% as opposed to 10% for* $I(\theta_t; V_t)$*,; see the differences between the cyan and pink bars in Fig.2B. Because having axonal gap junctions can only reformat information received from the dendrites, this suggests that placing gap junctions at this position may be a strategy the VS network uses to specifically improve the predictive capacity of its output encoding. Next, we investigate whether such improvement approaches the physical limits set by the input current or ego-rotation information, computed using the information bottleneck framework.*

6 - The article could be much more clear, and would definitely benefit from some streamlining. This work is a synergy of multiple approaches and research traditions - which is its strength. It however combines technical wording and explanations which make it confusing to readers who are not experts in all these fields (and I am clearly a member of this club). My specific suggestions are:

Shorten the introduction - it is very long and it is hard to understand what are the main contributions of the paper. Many parts of it could be moved to the results (e.g. description of the VS network)

We changed the structure of introduction and the initial two sections of result: we moved the details of the VS network to the Results. The introduction is now 2.5 pages long.

In the result section I (line 234-270), we also group all information theory definitions together with respect to the architecture of the VS network in Fig 1D. We hope this will clarify how they work with each other to frame the information bottleneck problem more clearly. The revisions outlined above also address some of these points that were brought up by R1 as well. We thank both reviewers for pushing us to streamline and clarify our manuscript; we think it helped quite a lot!
