# Supplementary material for: Maximally efficient prediction in the early fly visual system may support evasive flight maneuvers
Source: PLoS Comput Biol. 2021 May 20;17(5):e1008965. doi: 10.1371/journal.pcbi.1008965 (PMC8136689; doi:10.1371/journal.pcbi.1008965)
Supplement: Attachment — Submitted filename: Response_letter_Mar_2021.pdf [file pcbi.1008965.s011.pdf]

THE UNIVERSITY OF
CHICAGO

STEPHANIE E. PALMER, PH.D.
ASSOCIATE PROFESSOR
DEPARTMENT OF ORGANISMAL BIOLOGY AND ANATOMY
1027 EAST 57TH STREET, ANATOMY 104
CHICAGO, ILLINOIS 60637-1508
PHONE: 773-702-0771 FAX: 773-702-0037

March 10th, 2021

We thank both reviewers for the useful comments on our revised manuscript. Detailed responses are included below.

We prepared a github repository https://github.com/siwei-wang/VS_pred including all intermediate data and code we used to generate figures in the manuscript.

We provided two versions of the revised manuscript: one with highlights of all changes as well as a clean version.

Sincerely,

Stephanie Palmer, Siwei Wang, Axel Borst, and Idan Segev

Detailed responses to reviewers:

Reviewer #1

1) Line 62: point 1 is a run-on sentence.

We changed point 1 into two sentences:

The initial rotation is not stereotyped (i.e. purely 90° or on predetermined body axes as are saccadic flight turns [4]). It must be tuned to both the position of the looming threat and the animal's heading at the moment of detection.

2) Line 55: I think it would be good to add a few citations to back up this claim about previous work.

We added two citations to back up this claim:

1) Land M, Collett T. Chasing behaviour of houseflies (fannia canicularis). J Compara. 1974;89:331--357.
2) Fabian ST, Sumner ME, Wardill TJ, Rossoni S, Gonzalez-Bellido PT. Interception by two predatory fly species is explained by a proportional navigation feedback controller. Journal of The Royal Society Interface. 2018;15(147):20180466.

3) Figure S3 could use x and y axis labels and units. I'm guessing these are in degrees per second. Is this roll rotation or yaw or total? Which rotation types are quantified/plotted is something that could be clarified throughout.

We have revised Figure S3 accordingly to include labels on all axes. The units are degrees, since this is a readout of the rotation angle at that moment in time after the initiation of the escape behavior.

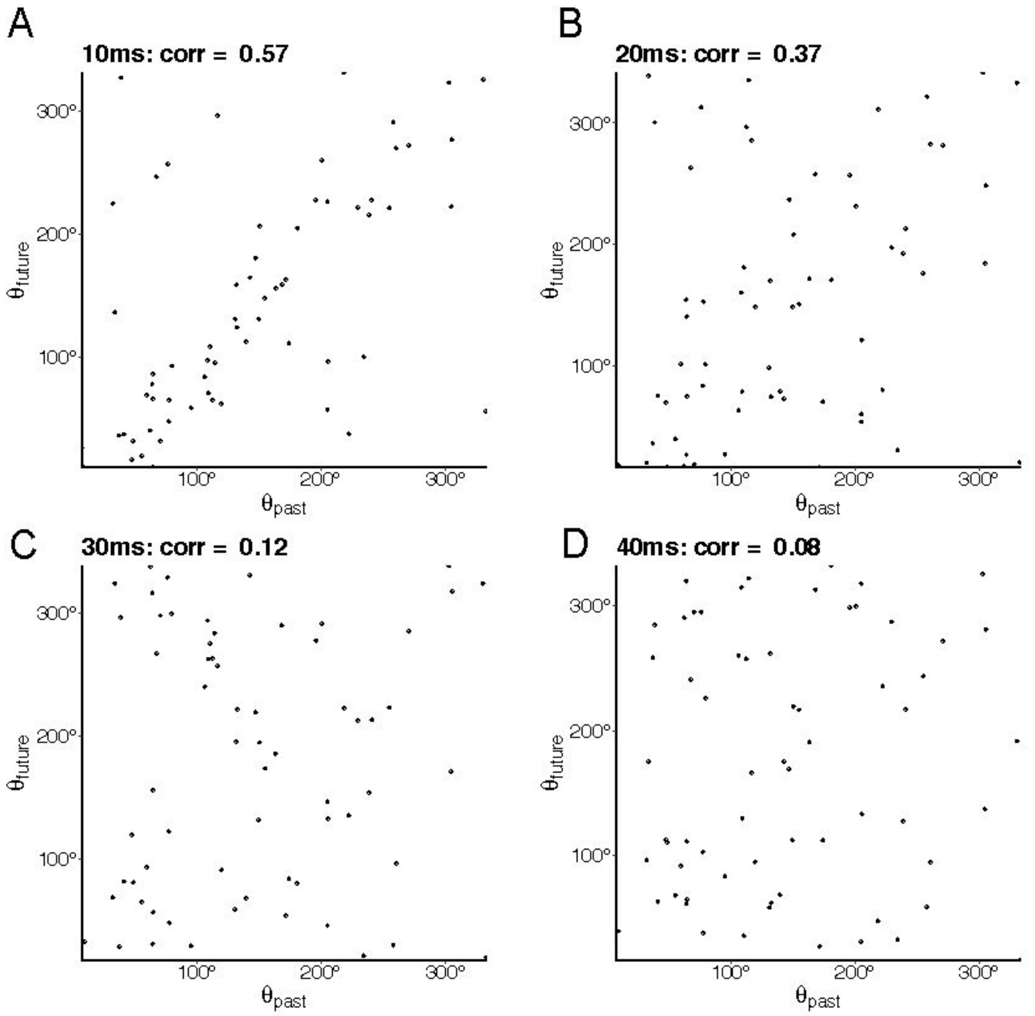

4) Line 285-288: Text does not quite match author's description of it in response to minor point 5, since it cites only Drews, not Matulis.

We cited Matulis2020 along with Drews in this revision (line 813-816 in the Reference section).

5) Line 561: I've only ever heard these referred to as 'campaniform sensilla', never 'campaniforms'.

Indeed, we should not invent new terminology here; we changed all the references of campaniforms to 'campaniform sensilla' here and later in this paragraph.

*The halteres may implement the above PI controller in a different ways, some of them may predict novel sensory pathways between the visual system and a visually gated motor neuron of wing steering muscles. The halteres were known as a multi-sensory integration circuit. They combine inputs from the eye, ocelli, antenna and themselves using multiple **campaniform sensilla** embedded on the stalk. One possibility is that the descending visual input recruits the Coriolis-sensitive sensilla. This activates the reflex loop consisting of dF2 campaniform sensilla and wing steering motor neurons and thus directly modify the firing phase of the tonic first basalar muscle (wB1), whose phase advance associates with increased wingbeat amplitude [88]. Alternatively, the visual prediction may use dF3, a **campaniform sensilla** that receives input independent from dF2, to recruit the phasic motor neuron, i.e. the second basalare motor neurons M.b2 and steering muscle w.B2. Little is known about these neurons other than that they are responsible for initiating these elevated wing kinematics (stroke amplitude and frequency [55, 56, 89]) which are necessary for evasive maneuvers. In the prediction paradigm, when the predictive visual information tunes the strength of haltere mechanosensory feedback [11], it indirectly activates these motor neurons via the haltere's connections to (M.b2) through electric synapses (fast enough for evasive maneuvers). Because of the lack of driver line targeting individual **campaniform sensilla** of the halteres, these are open questions for future investigation.*

6) Line 570: typo “the”?

Good catch! We changed it into “In the prediction paradigm …”

7) Line 630: Capitalize?

We capitalized it as “Ego-rotation for evasive flight maneuvers”

8) Line 736: “Blahut-Arimoto” should be capitalized here and later in paragraph.

We capitalized “Blahut-Arimoto” in all references in this paragraph:

*To choose K, we run the **Blahut-Arimoto** algorithm with a large (i.e. =100) to estimate the upper bound of mutual information, based on the observation that the information bottleneck reaches its optimum at $I(X;Y)$. Each **Blahut-Arimoto** estimation can take one week or longer in a multi-core cluster, so we only obtained the upper bounds for I_{future}, $I_{future}(\theta, \Delta t)$ and I_{past} for the entire VS network and the VS5-6-7 triplet.*

Reviewer #2:

1) I would encourage the Authors to include in the text some variant of their response to my question about the independence of the visual scene and the shape of the evasive trajectory (first question in the previous review). After all, this study connects statistics of stimuli to behavioral control, and many readers may wonder whether there is a link between the image of the obstacle/threat, and the evasive manuever.

This a good justification to include and we have added the following clarification to the Results:

In the original paper describing the escape trajectories of Drosophila, they found that escape trajectories remain consistent despite different expansion velocities and expansion angles of the visual threat. Therefore, the specific visual features of the threat were not important. These authors also used a large range of sizes for the spatially uniform visual threat. A visual threat as large in diameter as their maximum expansion angle (107°) is similar to a uniform object found in a natural scene, like a large patch of sky or other large, low-contrast object. Thus, we hypothesize that using a generic natural scene background will not significantly change our results.
